# Fluorination Effect for Highly Conjugated Alternating Copolymers Involving Thienylenevinylene-Thiophene-Flanked Benzodithiophene and Benzothiadiazole Subunits in Photovoltaic Application

**DOI:** 10.3390/polym12030504

**Published:** 2020-02-25

**Authors:** Lili An, Yubo Huang, Xu Wang, Zezhou Liang, Jianfeng Li, Junfeng Tong

**Affiliations:** 1 Key Laboratory for Utility of Environment- Friendly Composite Materials and Biomass in University of Gansu Province, School of Chemical Engineering, Northwest Minzu University, Lanzhou 730030, China; 2 School of Materials Science and Engineering, Lanzhou Jiaotong University, Lanzhou 730070, China; yubohuang94@163.com (Y.H.); 15235255485@163.com (X.W.); zezhouliang@hotmail.com (Z.L.); ljfpyc@163.com (J.L.); tongjunfeng139@163.com (J.T.); 3 CAS Key Laboratory of Bio-based Materials, Qingdao Institute of Bioenergy and Bioprocess Technology, Chinese Academy of Sciences, Qingdao 266101, China

**Keywords:** thienylenevinylene-thiophene-flanked benzodithiophene, benzothiadiazole, fluorination effect, photovoltaic application

## Abstract

Two two-dimensional (2D) donor–acceptor (D-A) type conjugated polymers (CPs), namely, PBDT-TVT-BT and PBDT-TVT-FBT, in which two ((*E*)-(4,5-didecylthien-2-yl)vinyl)- 5-thien-2-yl (TVT) side chains were introduced into 4,8-position of benzo[1,2-*b*:4,5-*b*ʹ]dithiophene (BDT) to synthesize the highly conjugated electron-donating building block BDT-TVT, and benzothiadiazole (BT) and/or 5,6-difluoro-BT as electron-accepting unit, were designed to systematically ascertain the impact of fluorination on thermal stability, optoelectronic property, and photovoltaic performance. Both resultant copolymers exhibited the lower bandgap (1.60 ~ 1.69 eV) and deeper highest occupied molecular orbital energy level (*E*_HOMO_, –5.17 ~ –5.37 eV). It was found that the narrowed absorption, deepened *E*_HOMO_ and weakened aggregation in solid film but had insignificant influence on thermal stability after fluorination in PBDT-TVT-FBT. Accordingly, a PBDT-TVT-FBT-based device yielded 16% increased power conversion efficiency (PCE) from 4.50% to 5.22%, benefited from synergistically elevated *V*_OC_, *J*_SC_, and *FF*, which was mainly originated from deepened *E*_HOMO_, increased *μ*_h_, *μ*_e_, and more balanced *μ*_h_/*μ*_e_ ratio, higher exciton dissociation probability and improved microstructural morphology of the photoactive layer as a result of incorporating fluorine into the polymer backbone.

## 1. Introduction

Of all the third generation solar cell technologies (i.e., organic solar cells, perovskite solar cells, and dye-sensitized solar cells, and so on), bulk-heterojunction (BHJ) polymer solar cells (PSCs) have attracted broad academic and industrial interests, because of attractive features including flexibility, lightweight, large area, low-cost production, and environmental friendliness [1,2,3,4,5,6,7,8,9,10,11,12,13,14,15,16,17,18,19]. The photovoltaic performance of BHJ PSCs was highly dependent on the semiconductor materials used as the photoactive layer. Thus, tremendous efforts in the past decades have been devoted to exploring excellent semiconducting materials, such as conjugated copolymers (CPs) and small molecules (SMs), and the use of various additives to modulate the morphology and optimization of processing techniques [20,21,22,23,24,25,26,27,28,29,30,31,32,33,34]. Recently, with the help of novel materials, improved morphology, much more profound understanding of the structure–performance correlation and the photon-to-electron conversion mechanism, and power conversion efficiencies (PCEs) for PSCs in the single-junction module of over 11% in fullerene systems [35] and over 16% in non-fullerene systems [36,37,38,39] were achieved. It has proved that constructing donor–acceptor (D-A) type CPs and/or SMs appears to be one of the most simplistic, promising, attractive, and successful strategies, since the alternating D-A conjugated blocks in the polymer backbone not only led to the reduction of the band gap by hybridizing the highest occupied molecular orbital (HOMO) with the lowest unoccupied molecular orbital (LUMO), but also enhanced the inter- and intra-molecular interactions directly governing the molecular ordering and π–π stacking for conjugated materials in the solid state. As a consequence, the molecular properties for D-A type semiconducting materials, such as frontier energy levels, absorption, dipole moment, planarity of the backbone, intermolecular interaction, charge transport characteristics, and morphology could be fine-modulated by judiciously selecting D and A units and properly combining them, assisted by adjusting the photo-induced intra-molecular charge transfer (ICT) effect [7,20,21,22,23,24,26,36,37,38,39,40,41]. In order to maximize the PCE of PSCs, it was of critical importance that, on one hand, enlarging the difference as much as possible between the *E*_HOMO_ of donor and *E*_LUMO_ of acceptor could obtain a larger open-circuit voltage (*V*_OC_), meanwhile the suitable *E*_LUMO_ paired with the electron acceptor was also juggled for charge separation and transport, while on the other hand, reducing the optical band gap toward harvesting more photons by up-shifting the *E*_HOMO_ or down-shifting the *E*_LUMO_ was anticipated to yield higher short-circuit current density (*J*_SC_) [7,8]. However, simultaneously obtaining both high *V*_OC_ and *J*_SC_ was difficult and needed a lot of trials for balancing energy levels. Consequently, making a thorough inquiry into the structure–property–performance relationship and constructing efficient CPs by way of choosing the appropriate D and A units still presented a major challenge for material researchers. 

Of the various CPs in the D-A system, one of the most promising and representative electron- donating building blocks, benzo[1,2-*b*:4,5-*b*′]dithiophene (BDT) has been widely focused on and garnered considerable interest due to its large planar conjugated backbone and, hence, better π–π stacking, suitable electron-donating ability, and easily functionalized 4,7-positions which were substituted by non-conjugated side chains, i.e., alkyl (R), alkyloxy (RO), alkylthio (RS), tri-*iso*- propylsilylethnyl (TMS), and conjugated ones, including alkylthienyl (RT), alkylthiothienyl (RST), 5-alkylthieno[3,2-*b*]thiophen-2-yl (RTT), 2,2′-bithiophen-2-yl (R2T), alkylphenyl (RPh), alkylfuran (RF), 2′-alkyl-2,2′-diphenyl-2-yl (RDiPh), alkylbenzothiophen-2-yl (RBT), 9,9-dialkylfluorene-2-yl (RFl), *N*-9-alkylcarbazole-2-yl (RCz), alkylthionaphthyl (RSN) and alkylthienylenevinylene thiophen-2-yl (TVT) groups, and so on [42,43,44,45,46,47,48,49,50,51,52,53,54,55,56,57,58,59,60,61,62,63,64]. Compared to their one-dimensional (1D) counterparts, two-dimensional (2D) photovoltaic polymers by vertically expanding their conjugated region could gain many unexpected characteristics [65,66,67,68,69]. Early in 2006, Hou et al. found 2D polythiophenes containing a bis(thienylenevinylene)-conjugated side chain (biTV-PTs) showing a broadened absorption, deepened *E*_HOMO_ compared to their counterpart P3HT, and particularly simultaneously enhanced *V*_OC_, *J*_SC_, fill factor (*FF*), and corresponding PCE were all improved in P3 [65]. They continuously introduced the RT-conjugated side chain into benzo[1,2-*b*:4,5-*b*′]difuran (BDF) to prepare 2D PBDF-TT-CF-T, exhibiting stronger absorption in the low-energy region, 0.23 eV lowered *E*_HOMO_ and 45-times elevated space-charge-limited-current (SCLC) hole mobility (*μ*_h_), and thus 0.15 V raised *V*_OC_ and 20% increased PCE were achieved relative to 1D PBDFTT-CF-O [70]. In the meantime, a 110 °C enhanced onset decomposition temperature (*T*_d_) in thermogravimetric analysis, red-shifted absorption peak and reduced optical bandgap ($E_{g}^{\mathrm{opt}}$), uninfluenced *E*_HOMO_, and the corresponding improved PCE from 3.06% to 5.00% were also observed in quinoxaline (Qx)-based CPs, when the RO side chain was substituted by conjugated RT [71]. Next, by introducing RT into BDT to enlarge the conjugated planarity of PTB7, a 2D PTB7-DT featuring the more pronounced red-shifted absorption (λ_max_ ≈ 708 nm), 0.34 eV downward *E*_HOMO_, 4-times higher *μ*_h_, and smaller domains and better miscibility when blended with PC_71_BM, was explored and applied in photovoltaic application, which enable a PTB7-DT:PC_71_BM-based device to obtain a PCE of up to 10.12% [72]. In 2016, Sun et al. studied the conjugation effect in dithieno[3′,2′:3,4;2′′,3′′:5,6]- benzo[1,2-*c*]oxadiazole (fDTBO)-based CPs, and found that there existed an 84 °C increased *T*_d_, 25 nm red-shifted maximum absorption peak, and 0.08 eV reduced $E_{g}^{\mathrm{opt}}$, 0.18 eV deepened *E*_HOMO_, and 10.7-fold higher SCLC mobility and thus 61% increased PCE from 2.80% to 4.52% than those of a 1D counterpart [73]. Meanwhile, 53% enhancement in PCE from 3.00% to 4.59% in 3,3′-(ethane-1,2- diylidene)bis(indolin-2-one) (ECI)-based wide bandgap CPs was realized after the conjugated alkylthienyl side chain was utilized, mainly benefiting from slightly 9 nm red-shifted absorption peak from 667 to 676 nm, 0.05 eV down-shifted *E*_HOMO_ from –5.46 to –5.51 eV, and 1.35-times increased hole mobility [74]. Afterward, replacing RO side chain of BDT with conjugated RTT, a remarkable improvement in PCE from 0.54% to 8.43% in the BDT-*alt*-bithiophene backbone was ascribed to the wide light absorption, higher electron and hole mobilities (*μ*_e_ and *μ*_h_) and balanced *μ*_e_/*μ*_h_, good phase separation of photoactive layer, and high exciton dissociation efficiency [75]. In the meantime, Chen et al. introduced two conjugated side chains into both BDT and fluorinated thieno[3,4-*b*]thiophene and developed a series of full-2D CPs, and found that simultaneously integrating the 2D-TT and 2D-BDT CPs exhibited remarkably efficient absorption of sunlight, improved π–π intermolecular interaction for efficient charge transport, and preferentially ordered microstructure, leading to increased *J*_SC_, *FF*, and hence 10.8% improved PCE from 8.10% to 8.98% [76]. It was crystal clear that the 2D conjugated side chain usually induced a red-shifted absorption to better match the solar spectrum, an improved π-orbital overlap between the conjugated polymer chains, and a higher charge mobility to reduce the energy loss aimed at theoretically acquiring the higher *J*_SC_ and *FF,* compared with 1D CPs. Evidently, these reported conjugated side chains mainly concentrated on the alkylthienyl and alkylphenyl. More importantly, one highly conjugated building block, thienylene-vinylene-thienylene or thienylenevinylene thiophene, linked via vinylene (bearing double-bond characteristic) between two thiophenes, which presented high charge-carrier mobility as result of the increased degree of coplanarity for the polymer backbone, was widely investigated and applied in organic thin-film transistors (OTFT) and selected as the main chain incorporated into the polymer backbone for solar cells [33,77,78,79,80,81,82,83]. However, selecting thienylenevinylene thiophene as the conjugated side chain attached onto backbone was overlooked to some extent and there were some limited examples [61,62,63]. In 2014, Kang et al. introduced an alkylthienylenevinylene thiophene (R-TVT) side group into BDT to construct BDTTVT and then copolymerized with dithienyl thieno[3,4-*c*]pyrrole-4,6-dione (DTTPD) to prepare PBDTTVT-DTTPD and found that the R-TVT side chain red-shifted the absorption peak, increased the absorption intensity, and slightly reduced bandgap, as well as creating a more advantaged microstructure of morphology, resulting in a 35.4% increased *J*_SC_ and thus 25.3% improved PCE from 4.82% to 6.04%, respectively, compared to control polymer PBDTT-DTTPD bearing alkylthienyl side group onto BDT [61]. Following this, the alkyloxy side chain onto BDT in PTB7 was replaced with alkylthiothienylenevinylene thiophene (RS-TVT) to develop a 2D CP PBT-TVT, and a greatly improved absorption ranged from 300 to 550 nm, reduced optical band gap from 1.63 to 1.53 eV, 0.06 down-shifted *E*_HOMO_, more ordered solid film stacking, and 2.22-times raised *μ*_h_ were observed, leading to synergistically increased *V*_OC_ and *J*_SC_ and corresponding 9.7% enhanced PCE from 7.41% to 8.13% as the result of prolonged conjugation of incorporating RS-TVT on BDT [62]. Recently, our group developed a 4,5-didecyl- thienylenevinylene thiophene (DR-TVT) modified BDT-containing CPs and investigated the effect of electron withdrawing [63]. As for the various electron-acceptor units utilized, benzo[*c*][1,2,5]- thiadiazole (BT) and its derivatives were considered as the stronger electron-withdrawing moieties and tremendous BT-based semiconducting materials have been focused on, because of subtly integrating its electron accepting property and its ability to adopt the quinoid structure, easy availability, and modification [7,20,21,24,28,35,37,41,42,47,49,50,58,63,64,73,84,85,86,87,88]. 

**Scheme 1:**
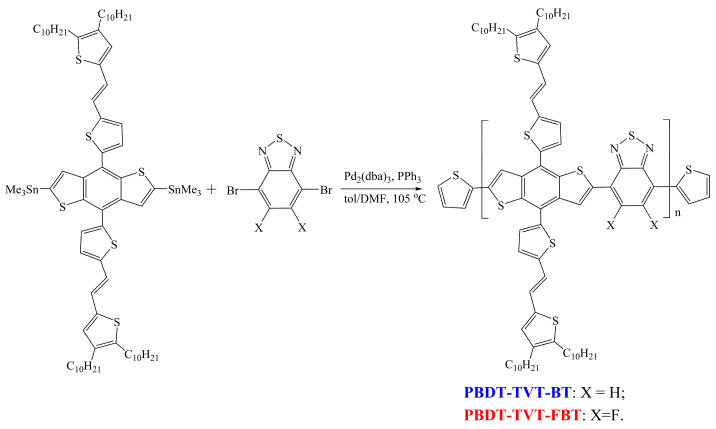
Synthetic routes of the studied highly conjugated copolymers (CPs) PBDT-TVT-BT and PBDT-TVT-FBT.

Prompted by the abovementioned considerations, herein, two ((*E*)-(4,5-didecylthien-2-yl)- vinyl)-5-thien-2-yl (TVT)-conjugated side chains were introduced into the 4,8-positions of BDT, and this building block was further copolymerized with BT and/or 5,6-difluoro-substituted-BT (FBT) to construct 2D D-A type alternating CPs, namely, PBDT-TVT-BT and PBDT-TVT-FBT, respectively, as shown in Scheme 1. The effects of fluorination on thermal stability, their absorption spectra, energy levels, aggregation, charge mobility, exciton dissociation rate, the morphology for photoactive layer, and thus photovoltaic performance were systematically studied. The fluorination could down-shift the *E*_HOMO_, weaken the solid aggregation, and improve the microstructure of the active layer. As a consequence, 16% increased PCE from 4.50% to 5.22% was achieved after incorporating fluorine into BT in PBDT-TVT-FBT-based device.

## 2. Materials and Methods

### 2.1. Characterization 

^1^H NMR spectra; melting points test; C, H, and N elemental analyses; TGA analyses; molecular weights of polymer; UV–Vis absorption; electrochemical cyclic voltammetry (CV); thin film X-ray diffraction (XRD); atomic force microscopy (AFM); and transmission electron microscopy (TEM) images were obtained according to our reported methods [86,87,88,89]. 

### 2.2. Materials

All reagents were of reagent grade and purchased from Sigma-Aldrich (Shanghai) Co. (Shanghai, China), Acros (Geel, Belgium,), J&K scientific (Beijing, China) and TCI (Shanghai) Chemical Co. (Shanghai, China) and used without further purification, unless otherwise noted. The interlayer material 3,3′-(1,3,8,10-tetraoxoanthra[2,1,9-*def*:6,5,10-*d*′*e*′*f*′]diisoquinoline-2,9(1*H*,3*H*,8*H*,10*H*)diyl)bis(*N*,*N*-dimethylpropan-1-amine oxide) (PDINO) and 4,7-dibromobenzo[*c*][1,2,5]thiadi- azole (BTBr_2_) were purchased from Derthon Optoelectronic Materials Science Technology Co. Ltd. (Shenzhen, China). Bistin 2,6-bis(trimethyltin)-4,8-bis[5-((*E*)-2-(4,5-didecylthien-2-yl)vinyl)-5-thien- 2-yl]benzo[1,2-*b*:4,5-*b*’]dithiophene (BDT-TVTSn) and fluorinated 4,7-dibrom-5,6-difluorobenzo[*c*]- [1,2,5]thiadiazole (FBTBr_2_) were prepared according to the reported references [87,90]. All key comonomers were identified by NMR before utilization in the supporting information.

### 2.3. Polymer Preparation

Two TVT-containing alternating CPs were prepared following these procedures: carefully purified bistin BDT-TVTSn and dibromo-monomer (BTBr_2_ and FBTBr_2_) were fully dissolved into 6 mL degassed dry toluene and 0.8 mL DMF in a 25 mL two-neck round-bottom flask under Ar. The mixture was bubbled with Ar for another 20 min to remove O_2_. Thereafter, Pd_2_(dba)_3_ (1.4 mg) and P(o-tolyl)_3_ (2.3 mg) were quickly added to the mixture in one portion and the solution was bubbled with Ar for additional 20 min. The mixture was then vigorously refluxed for 48 h under Ar, followed by the subsequent addition of 2-tri(butylstannyl)thiophene and 2-bromothiophene at an interval of 8 h in order to finish ending-capping. After additional reflux at 8 h, the mixture was poured into 300 mL MeOH. The precipitate was collected by filtration and the crude polymer was subjected to Soxhlet extraction successively with MeOH, acetone, hexane, and toluene. Finally, the toluene fraction was condensed to approximately 6 mL and precipitated into MeOH. The black solid was collected and completely dried under vacuum overnight to obtain the target material with a yield of 67.8%~69.6%.

#### 2.3.1. Synthesis of Poly[4,8-bis[5-((E)-2-(4,5-didecylthien-2-yl)vinyl)-5-thien-2-yl]benzo[1,2-b:4,5-b’]di-thiophene-2,6-diyl-alt-benzo[c][1,2,5]thiadiazole-4,7-diyl] (PBDT-TVT-BT)

BDT-TVTSn (163.8 mg, 0.112 mmol) and BTBr_2_ (32.9 mg, 0.112 mmol) were used to prepare the PBDT-TVT-BT on the basis of the foregoing synthesis procedure. About 96.0 mg of PBDT-TVT-BT was obtained in 67.8% yield as a black solid. Number-average molecular weights (*M*_n_) = 17.4 kDa, polydisperse index (PDI = *M*_w_/*M*_n_) = 1.90. Anal. Calcd. for C_76_H_98_N_2_S_7_: C, 72.21%; H, 7.81%; N, 2.22%. Found: C, 72.11%; H, 7.69%; N, 2.33%. 

#### 2.3.2. Synthesis of Poly[4,8-bis[5-((E)-2-(4,5-didecylthien-2-yl)vinyl)-5-thien-2-yl]benzo[1,2-b:4,5-b’]di-thiophene-2,6-diyl-alt-5,6-difluorobenzo[c][1,2,5]thiadiazole-4,7-diyl] (PBDT-TVT-FBT)

BDT-TVTSn (163.8 mg, 0.112 mmol) and FBTBr_2_ (46.46 mg, 0.112 mmol). About 101.3 mg of fluorinated PBDT-TVT-FBT was collected as a black solid (yield: 69.6%). *M*_n_ = 18.3 kDa, PDI = 2.10. Anal. Calcd. for C_76_H_96_F_2_N_2_S_7_: C, 70.21%; H, 7.44%; N, 2.15%. Found: C, 70.01%; H, 7.39%; N, 2.25%.

### 2.4. Fabrication and Characterization of PSCs

The fabrication process of the solar cells testing the TVT-containing alternating CPs was prepared according to our reported reference [91].

### 2.5. Hole-Only Device Fabrication and Measurement

The hole and electron mobilities were measured from the *J*–*V* curves obtained under dark current in light of the steady-state SCLC method described in our reported reference [86,89,92]. 

## 3. Results and Discussion

### 3.1. Molecular Design, Synthesis, and Characterization 

**Figure 1:**
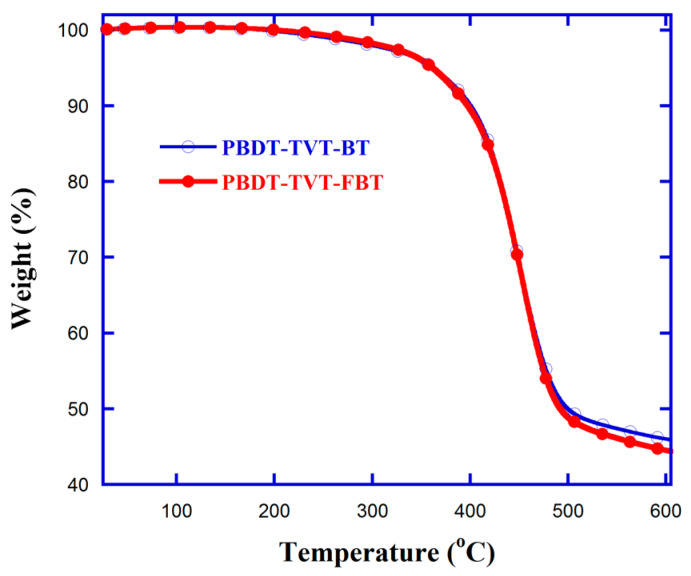
TG curve of the highly conjugated donor–acceptor (D-A) type copolymers PBDT-TVT-BT and PBDT-TVT-FBT.

Bistin BDT-TVTSn and fluorinated dibromide FBTBr_2_ were prepared according to the reported method [87,90]. The synthetic route for FBTBr_2_ has been described in the Supporting Information. These utilized key comonomers were identified by ^1^H/^13^C NMR (Figures S1–S4) and elemental analyses. As outlined in Scheme 1, the resultant highly conjugated D-A type 2D CPs PBDT-TVT-BT and PBDT-TVT-FBT were synthesized via typical Stille polymerization strategy [93], and the further post-processing and purification steps were accomplished in accordance with the previously reported method [89]. Finally, the fraction dissolved in toluene was recovered by re-precipitating in MeOH, and then dried under vacuum overnight so as to remove the residual solvents. Note that the studied highly conjugated polymers PBDT-TVT-BT and PBDT-TVT-FBT were acquired as black solids with yields of 67.8% and 69.6%, respectively. These highly conjugated copolymers exhibited limited solubility in chloroform due to introducing the ((*E*)-(4,5-didecyl- thien-2-yl)vinyl)-5-thien-2-yl side chain containing double-bond characteristic resulting in increased coplanarity of the polymer backbone, but sufficient solubility in hot chlorobenzene (CB) and *o*-dichlorobenzene (oDCB) solution. The measurements for gel permeation chromatography (GPC) depicted that *M*_n_ and PDIs values were found to be 17.4 kDa and 1.90 for PBDT-TVT-BT and 18.3 kDa and 2.10 for PBDT-TVT-FBT, respectively (Table S1), implying that the effect of molecular weights on the optoelectronic and photovoltaic behavior scarcely existed. Furthermore, the decomposed temperatures (*T*_d_, 5% weight-loss temperature) in thermogravimetry analysis (TGA) for PBDT-TVT-BT and PBDT-TVT-FBT were all to be 362 °C (Figure 1), suggesting that these highly conjugated 2D alternating copolymers possessed enough thermo-stability in the photovoltaic application and that fluorination in 2D D-A type TVT-containing CPs never produced an impact on the thermo-stability.

### 3.2. Optical Property 

**Figure 2:**
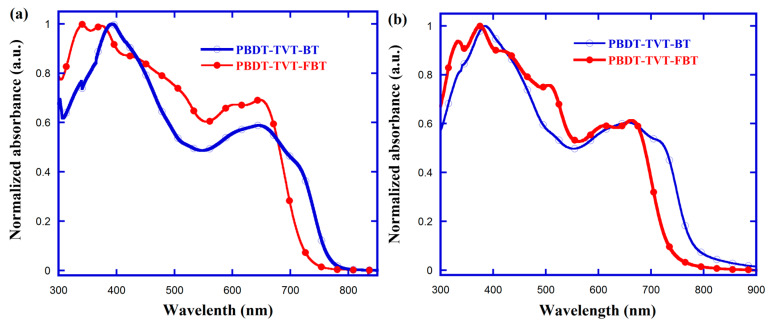
Normalized UV–Vis absorption spectra of the highly conjugated copolymers PBDT-TVT-BT and PBDT-TVT-FBT in diluted chlorobenzene (CB) (**a**) and thin-film state (**b**).

The normalized UV–Vis absorption spectra in CB solution (ca. 10 *μ*M) and as solid films were examined to investigate the impact of fluorination in highly conjugated TVT-containing alternating CPs on photophysical characteristics. The absorption spectra for these TVT-containing CPs are shown in Figure 2 and the key photophysical parameters are also listed in Table 1. Apparently, both copolymers featured broad visible absorption with clear bands, that is, one relatively stronger absorption situated at high-energy region could be assigned to the π–π* transition of BDT units bearing the conjugated TVT side chains, whereas the absorption band at the low-energy range was attributed to intramolecular charge effect between BDT units bearing the TVT-conjugated side chain and electron-deficient BT/FBT moiety from the polymer backbone, as well as one shoulder peak related with the aggregation behavior [20,44,61,62]. We noted that when the side chain of BDT was an alkyloxy and/or alkylthienyl in these BT-based CPs, the intensity of the high-energy absorption band was lower than that of the low-energy absorption band [42,93], and when the side chain was enlarged to bithienyl and terthienyl, the intensity of the high-energy absorption band increased and was higher than that of the low-energy absorption band [44]. However, when the BDT core was expanded horizontally to dithieno[2,3-*d*:2′,3′-*d*′]benzo[1,2-*b*:1,2-*b*′]dithiophene (DTBDT), the intensity of the high-energy absorption band decreased and was dramatically lower than that in the low-energy region [94]. In detail, the 9 nm blue-shifted value (383–392 nm) for π–π* peak, 15 nm (from 648 to 663 nm) and only 5 nm (from 712 to 717 nm) red-shifted values for corresponding ICT and shoulder peaks were observed, whereas the 8 and 0 nm blue-shifted values and a newly emerged peak at 508 nm due to the π–π* transition, both 15 nm red-shifted values (from 600 to 615 nm and from 648 to 663 nm) for ICT and shoulder peaks were also observed in fluorinated PBDT-TVT-FBT, ongoing from solution to film state. The blue-shifted absorption spectra were observed after fluorination both in CB solution and solid film, which were identical with the results observed in DTBDT-TIPS-*alt*-BT and naphthobisthiadiazole-*alt*-bithiophene system [88,95]. Furthermore, the onsets of the optical absorptions for PBDT-TVT-BT and PBDT-TVT-FBT films were found to be 775 nm ($E_{g}^{\mathrm{opt}}$ = 1.60 eV) and 735 nm ($E_{g}^{\mathrm{opt}}$ = 1.69 eV), respectively. Interestingly, the absorption intensity ranging from 450 to 700 nm in CB solution exhibited remarkable enhancement. In order to more precisely judge the sunlight-harvesting abilities of conjugated materials, the molar absorption coefficients (*ε*) in CB solution were further measured, as shown in Figure S5. The absorption coefficient values for PBDT-TVT-BT and PBDT-TVT-FBT were found to be 17,789 and 17,048 L mol^−1^ cm^−1^, respectively, indicating the fluorination produced little influence on absorption coefficient [86,96]. 

**Figure 3:**
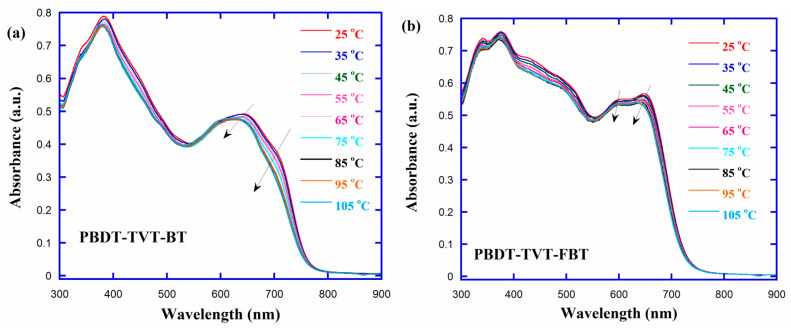
Temperature-dependent absorption (TD-Abs) spectra for the highly conjugated copolymers (**a**) PBDT-TVT-BT and (**b**) PBDT-TVT-FBT.

Systematic spectroscopic studies were carried out to probe the presence of polymer aggregates in CB solution by elevating the temperature to dissolve the crystallites. Since the higher temperature could support the energy needed to increase the molecular motion which led to breaking the aggregates to form isolated chains [44,86−89], the temperature-dependent absorption (TD-Abs) spectra of PBDT-TVT-BT and PBDT-TVT-FBT in CB solution (ca. 10 *μ*M), ranging from 25 to 105 °C with a 10 °C interval, were measured to examine the difference presence or absence of fluorination in these highly conjugated polymers containing TVT side chain on aggregation in solution. As presented in Figure 3, the absorption for both copolymers showed a similar change trend, that is, the ICT peak and shoulder peak were all hypsochromically shifted and the corresponding absorption intensity gradually decreased when the temperature was elevated from room temperature to 105 °C. In detail, during the elevating temperature process, the blue-shifted values (*Δ*λ) and the decreased absorption intensity (*ΔI*) for ICT absorption peak were 21 nm (from 648 to 627 nm) and 2.8% for PBDT-TVT-BT, 6 nm (from 602 to 596 nm) and 3.0% for PBDT-TVT-FBT, respectively. In view of the observed small discrepancies, we could infer that fluorination played little impact on the aggregation for these studied highly conjugated polymers in diluted CB solution [44,87,97].

### 3.3. X-ray Diffraction Analysis

**Figure 4:**
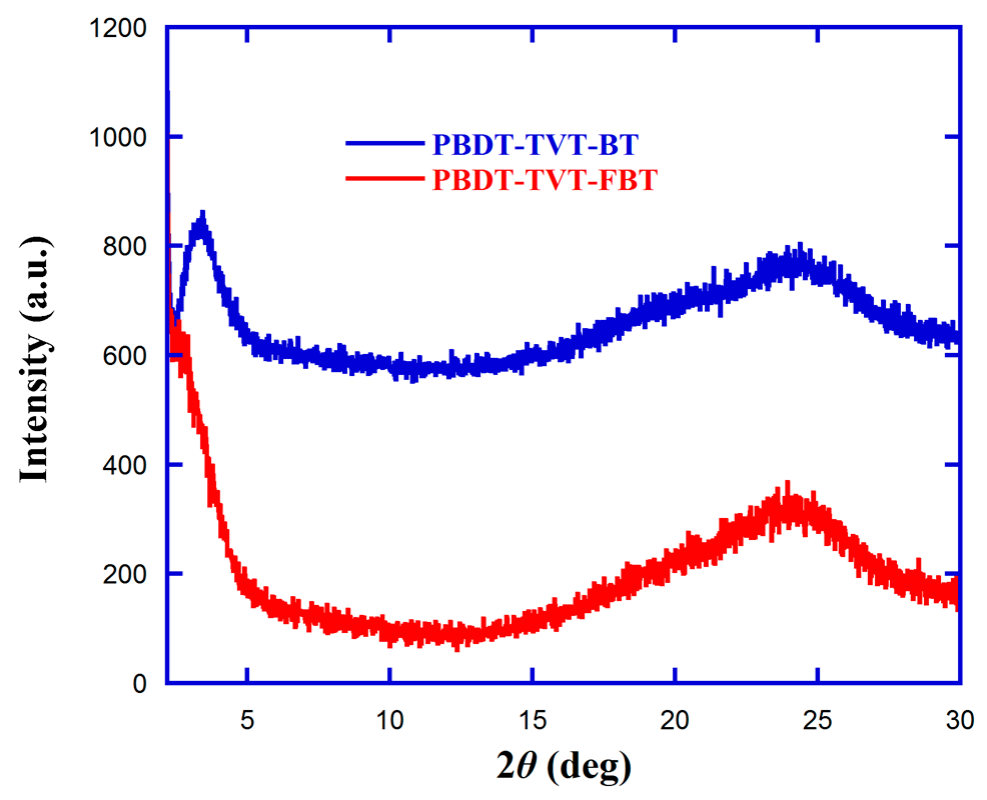
X-ray patterns of PBDT-TVT-BT and PBDT-TVT-FBT film on glass.

In order to further investigate the impact of fluorination of highly conjugated copolymers on crystallinity and molecular packing in solid-film state, X-ray diffraction (XRD) analyses of the pristine polymers were used. Note that the XRD spectra for polymer thin films were tested by drop-casting their corresponding CB solution onto the glass substrate. Figure 4 shows the XRD spectra of the studied copolymers PBDT-TVT-BT and PBDT-TVT-FBT. The *d*-spacing was obtained according to Bragg’s equation, *n*λ = 2*d*_hkl_sin*θ*, where λ is the radiation wavelength, *d*_hkl_ is the specific lattice spacing, and *θ* is the diffraction angle [44,87]. For PBDT-TVT-BT, there existed two distinct diffraction peaks, i.e., 2*θ* = 3.36° in the low-angle region and 2*θ* = 24.21° in the high-angle region, which corresponded to an inter-chain lamellae packing spacing of 26.26 Å and π–π stacking distance of 3.67 Å, respectively. Similarly, one indistinct diffraction peak situated at approximately 2*θ* = 2.75° and the other distinct diffraction peak at 2*θ* = 24.13° after fluorination were also observed, corresponding to 32.09 and 3.68 Å, respectively. These greatly enhanced the inter-chain lamellae packing distance, and similar π–π stacking suggested that fluorination weakened the solid aggregation and molecular ordered structure in fluorinated PBDT-TVT-FBT. 

### 3.4. Electrochemical Property 

**Figure 5:**
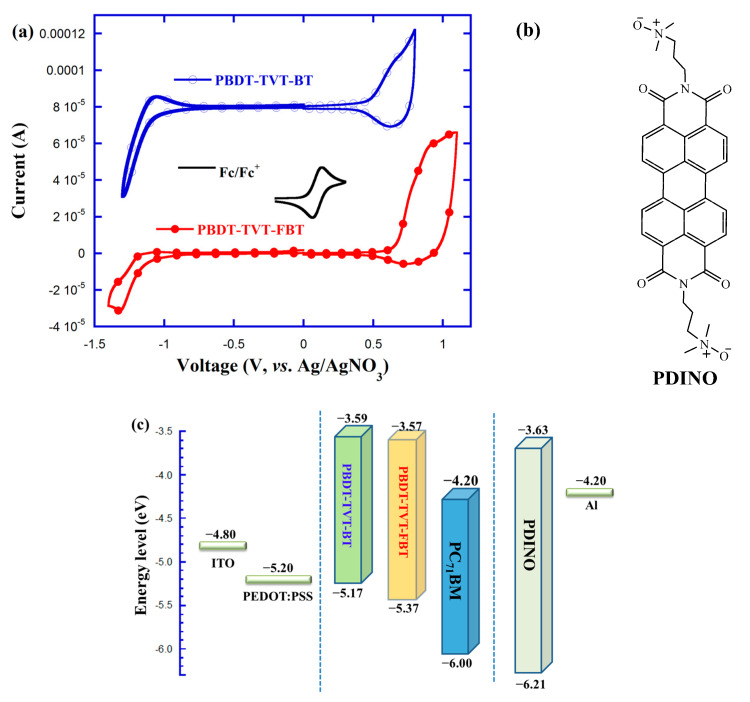
Cyclic voltammetry (CV) curves for the studied copolymers PBDT-TVT-BT and PBDT-TVT-FBT (**a**), chemical structure of PDINO (**b**), and the energy levels schematic diagram for the related materials utilized in polymer solar cells (PSCs) (**c**).

It was important to get the electronic structure of the semiconducting conjugated polymers because they have implications toward electronic and photovoltaic devices. Cyclic voltammetry (CV) method was applied to clarify the influence of fluorination on the electrochemical properties. As exhibited in Figure 5a, both copolymers presented clear oxidation onset potential ($\varphi_{\mathrm{ox}}^{\mathrm{onset}}$) and reduction onset potential ($\varphi_{\mathrm{red}}^{\mathrm{onset}}$). The corresponding *E*_HOMO_ and *E*_LUMO_ were calculated from their $\varphi_{\mathrm{ox}}^{\mathrm{onset}}$ and $\varphi_{\mathrm{red}}^{\mathrm{onset}}$, respectively. The corresponding $\varphi_{\mathrm{ox}}^{\mathrm{onset}}$ and $\varphi_{\mathrm{red}}^{\mathrm{onset}}$ values were found to be 0.47 and –1.11 V for PBDT-TVT-BT, and 0.67 and –1.13 V for PBDT-TVT-FBT, respectively (Table 1). Note that the CV curve was recorded with regard to the potential of the standard Ag/AgNO_3_ electrode, which was calibrated by the ferrocene-ferrocenium (Fc/Fc^+^) redox pair, and the *ϕ*_1/2_ of the Fc/Fc^+^ redox pair was 0.10 V vs. Ag/AgNO_3_ electrode. Supposing that the redox potential for Fc/Fc^+^ was −4.80 eV in relation to a vacuum energy level, thus the *E*_HOMO_, *E*_LUMO_, and the electrochemical bandgap ($E_{g}^{\mathrm{ec}}$) were estimated from the formula *E*_HOMO_ = –e($\varphi_{\mathrm{ox}}^{\mathrm{onset}}$ + 4.70), *E*_LUMO_ = –e($\varphi_{\mathrm{red}}^{\mathrm{onset}}$ + 4.70), and $E_{g}^{\mathrm{ec}}$ = *E*_LUMO_ – *E*_HOMO_ (eV), respectively [86]. The *E*_HOMO_, *E*_LUMO_, and $E_{g}^{\mathrm{ec}}$ values were calculated to be –5.17, –3.59, and 1.58 eV for PBDT-TVT-BT and –5.37, –3.57, and 1.80 eV for fluorinated PBDT-TVT-FBT, respectively. As depicted in Figure 5c, the schematic diagram related with the energy levels for the resultant CPs, PC_71_BM and other related materials in the PSCs was used to make a better comparison. The chemical structure of PDINO is given in Figure 5b. We can see that a 0.20 down-shifted *E*_HOMO_ value was achieved after introducing fluorine into the polymer backbone, which was helpful to acquire the higher *V*_OC_ theoretically, because the *V*_OC_ was increased when the difference between *E*_HOMO_ of donor and *E*_LUMO_ of acceptor was enlarged. Meanwhile, the difference between *E*_LUMO_ of donor and *E*_LUMO_ of PC_71_BM was 0.61 and 0.63 eV, respectively, indicating there existed enough driving force to facilitate an exciton to split into free charge [8,86].

### 3.5. Theoretical Calculation

**Figure 6:**
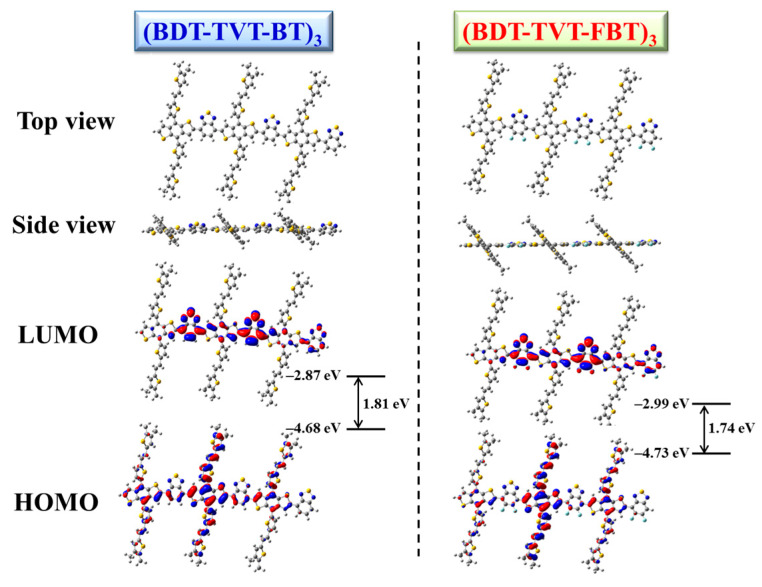
The optimized geometries and molecular orbital surfaces of the highest occupied molecular orbital (HOMO) and lowest unoccupied molecular orbital (LUMO) for the trimer model compound of PBDT-TVT-BT and PBDT-TVT-FBT.

The theoretical calculation was performed to predict the electronic properties and energy levels for the studied highly conjugated copolymers by utilizing the density functional theory (DFT) with the B3LYP/6-31G* basis set (Gaussian 09) [98]. In order to reduce the calculation cost, four decyl side chains from BDT-TVT were replaced by four methyl groups, and the studied polymer was simplified to a trimer. The optimized molecular geometries and frontier molecular orbitals are illustrated in Figure 6. The HOMO orbitals of the two studied polymers are primarily delocalized across the whole conjugated main chain and conjugated TVT side chain, while the LUMO orbitals are preferentially distributed in electron-deficient BT or FBT, suggesting the existence of a distinct ICT effect from BDT-TVT to BT or FBT. The calculated values of *E*_HOMO_, *E*_LUMO_, and bandgap were −4.68, −2.87, and 1.81 eV for PBDT-TVT-BT and −4.73, −2.99, and 1.74 eV of PBDT-TVT-FBT, respectively. After introducing fluorine into the polymer backbone in PBDT-TVT-BT, the decreased *E*_HOMO_ was found to be helpful to enhance the *V*_OC_, this alteration agreed with the results obtained from CV testing. Additionally, the impact of fluorination on the planarity of the polymer backbone was further evaluated by comparing the adjacent dihedral angle. The dihedral angles *θ*_1_ and *θ*_2_ between BDT and the conjugated TVT side group, and the dihedral angles *θ*_3_ and *θ*_4_ between BDT-TVT and BT and/or FBT moiety are listed in Table 2. It can be seen that such small variations of dihedral angles demonstrated that the fluorination scarcely improved the polymer molecular planarity, when the fluorine atoms were introduced into the polymer backbone in PBDT-TVT-FBT.

### 3.6. Photovoltaic Properties 

Photovoltaic properties were measured to gain insight into the effect of fluorination in highly conjugated polymers, by employing the device configuration of ITO/PEDOT:PSS/PBDT-TVT-BT or PBDT-TVT-FBT:PC_71_BM/PDINO/Al, in which the photosensitive layer was prepared by spin-coating the mixed solution involving PBDT-TVT-BT/PBDT-TVT-FBT and PC_71_BM, and the ultrathin interfacial layer (spinning from 1 mg L^–1^ methanol solution of PDINO) was applied as the cathode-modified interlayer [91]. The optimization processes for photovoltaic performance involved D/A ratio screening and 1,8-diiodioctane (DIO) processing additive, and the corresponding fabrication processes are listed in Supporting Information (Figure S6 and Table S2). When the D/A ratio was changed from 1:1 to 1:1.5 and then to 1:2, PBDT-TVT-BT-based devices exhibited unchanged *V*_OC_ of 0.74 V, first increased then decreased *J*_SC_ (from 8.95 to 9.29, then to 9.09 mA cm^−2^) and *FF* (from 49.99% to 56.67%, then to 53.71%) and thus it first increased, then decreased PCE (from 3.31% to 3.89%, then to 3.61%), whereas fluorinated PBDT-TVT-BT-based devices showed almost unchanged *V*_OC_ between 0.79 and 0.78 V, gradually increased *J*_SC_ (from 8.89 to 9.44, then to 9.46 mA cm^−2^) and first increased then decreased *FF* (from 55.71% to 58.67%, then to 56.19%) and thus first increased then decreased PCE (from 3.91% to 4.38%, then to 4.15%). Consequently, the optimal D/A ratios for PBDT-TVT-BT and PBDT-TVT-FBT were all to be 1:1.5. It was noted that the varied *FF* values were certified by the corresponding first increased then decreased shunt resistance (*R*_SH_*,* from 427.38 to 819.22, then to 790.33 Ω cm^2^ for PBDT-TVT-BT and from 978.67 to 1096.98, then to 981.93 Ω cm^2^ for PBDT-TVT-FBT) and first decreased, then increased series resistance (*R*_S_, from 17.53 to 13.63, then to 16.37 Ω cm^2^ for PBDT-TVT-BT and from 13.02 to 11.37, then to 12.73 Ω cm^2^ for PBDT-TVT-FBT). Apparently, the PBDT-TVT-BT-based device exhibited the best PCE of 3.89%, with a *V*_OC_ of 0.74 V, a *J*_SC_ of 9.29 mA cm^−2^, and an *FF* of 56.67%, while the fluorinated PBDT-TVT-FBT-based device shown a raised PCE of 4.38%, with the simultaneously increased *V*_OC_ of 0.79 V, *J*_SC_ of 9.44 mA cm^−2^, and *FF* of 58.67%. In the meantime, the EQE curves with different D/A ratios also agreed with *J*_SC_ in Figure S6. 

In order to maximize the photovoltaic performance, 3% DIO was selected as the solvent additive to optimize the morphology of the photosensitive layer, because processing solvent additive was helpful to form a more ordered and nano-scaled bicontinuous interpenetration network structure, which can not only support the abundant D/A interfaces facilitating the light-induced exciton to split into the free charge carriers but also provide the desired charge transport pathways [8,89]. As can be seen Figure S6 and Table S2, a 15.68% increment in PCE (from 3.89% to 4.50%) for the PBDT-TVT-BT-based device was observed, profiting from a joint 8.07% enhancement in *J*_SC_ (from 9.89 to 10.04 mA cm^−2^) and 6.88% rise in *FF* (from 56.67% to 60.57%). Similarly, a 19.18% elevation in PCE (from 4.38% to 5.22%) after introducing fluorine atoms into the polymer backbone in the PBDT-TVT-FBT-based device was found, chiefly benefiting from 11.76% enhanced *J*_SC_ (from 9.44 to 10.55 mA cm^−2^) and 8.13% up-shifted *FF* (from 58.67% to 63.44%) even with a slightly decreased *V*_OC_ (from 0.79 to 0.78 V). These increased *FF* values were also confirmed by the increased *R*_SH_ (from 819.22 to 1044.10 Ω cm^2^ for PBDT-TVT-BT and from 1096.98 to 1560.89 Ω cm^2^ for PBDT-TVT-FBT) and decreased *R*_S_ (from 13.36 to 11.33 Ω cm^2^ for PBDT-TVT-BT and from 11.37 to 9.17 Ω cm^2^ for PBDT-TVT-FBT). 

**Figure 7:**
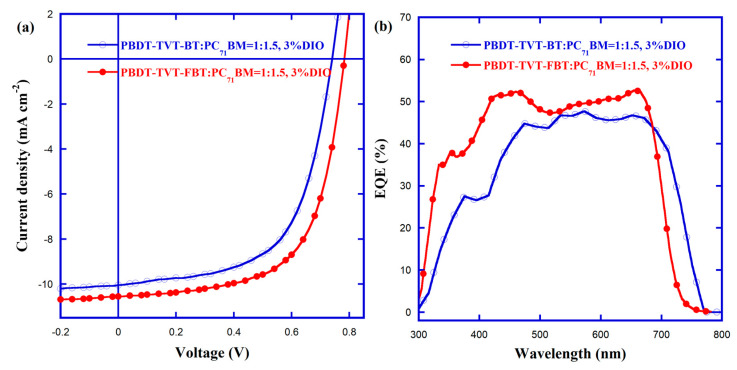
The optimal *J*–*V* curves (**a**) and EQE spectra (**b**) for PSCs based on PBDT-TVT-BT and PBDT-TVT-FBT.

The best *J–V* curves and corresponding external quantum efficiency (EQE) spectra are presented in Figure 7, and the best PV data are listed in Table 3. When introducing fluorine into the electron-withdrawing BT, a 16% increased PCE (from 4.50% to 5.22%) was found, which was benefited from a synergistic 5.41% increased *V*_OC_ (from 0.74 to 0.78 V), 5.08% enhanced *J*_SC_ (from 10.04 to 10.55 mA cm^−2^), and 4.74% elevated *FF* (from 60.57% to 63.4%). It was noted that these synergistically increased *V*_OC_, *J*_SC_ and *FF* values also corresponded to the former lowered *E*_HOMO_ (from −5.17 to −5.37 eV), enhanced EQE spectra in the high-energy range (Figure 7b), and increased *R*_SH_ (from 1044.10 to 1560.89 Ω cm^2^) and decreased *R*_S_ (from 11.33 to 9.17 Ω cm^2^), respectively [8,86,87,99]. Furthermore, the integrated *J*_SC_ values calculated from the EQE curves were found to be 9.94 and 10.35 mA cm^−2^ for the optimal PBDT-TVT-BT:PC_71_BM and PBDT-TVT-FBT:PC_71_BM-based devices, respectively, which were all in the range of tolerated error (< 5%) compared with *J*_SC_ values obtained from *J–V* measurements, demonstrating these *J*_SC_ values were reliable.

### 3.7. Exciton Dissociation and Charge Mobility 

**Figure 8:**
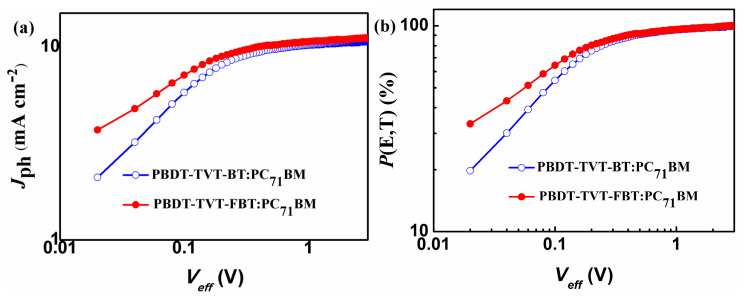
*J*_ph_ versus *V*_eff_ (**a**) and *P*(E,T) (**b**) versus *V*_eff_ curves for the optimized PBDT-TV-BT:PC_71_BM and PBDT-TVT-FBT:PC_71_BM-based devices.

To probe into the effect of fluorination in the highly conjugated copolymers on photocurrent properties, the photocurrent density (*J*_ph_) versus effective voltage (*V*_eff_) and corresponding exciton dissociation rate (*P*(E,T)) versus effective voltage (*V*_eff_) curves of the PSCs are displayed in Figure 8a,b, respectively. *J*_ph_ can be acquired from the formula: *J*_ph_ = *J*_light_ – *J*_dark_, where *J*_light_ and *J*_dark_ stand for current density under illumination and dark condition, respectively. *V*_eff_ is obtained from the equation *V*_eff_ = *V*_0_ − *V*_appl_, *V*_0_ represents the voltage when *J*_light_ is equal to *J*_dark_ and *V*_appl_ is the applied voltage. Furthermore, *P*(E,T) could be determined via the equation: *P*(E,T) = *J*_ph_/*J*_sat_, where *J*_sat_ is the saturated current density [64,91,99]. At *V*_eff_ = 3 V, the *J*_sat_ values of the photovoltaic devices were 10.68 mA cm^−2^ for PBDT-TVT-BT and 11.10 mA cm^−2^ for PBDT-TVT-FBT, respectively. Consequently, the corresponding *P*(E,T) values were 94.04% and 95.06%, suggesting the fluorination in PBDT-TVT- FBT:PC_71_BM-based device could facilitate exciton dissociation and reduce recombination, which can in part account for the obtained relatively higher *J*_SC_ and *FF*.

**Figure 9:**
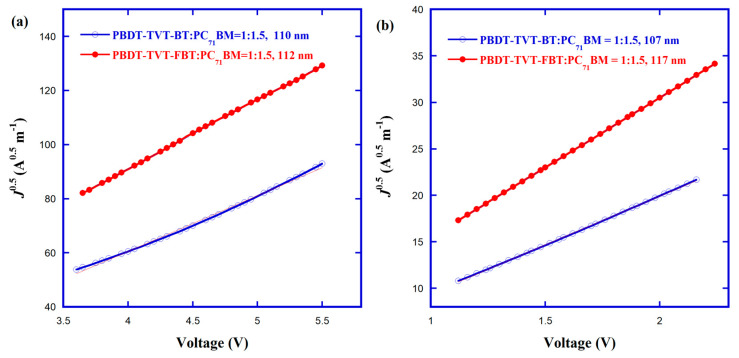
Fitting *J*^1/2^*–V* characteristics of hole-only (**a**) and electron-only (**b**) devices for PBDT-TV-BT:PC_71_BM and PBDT-TVT-FBT:PC_71_BM.

The vertical hole and electron transport properties were examined via hole-only and electron- only devices utilizing the corresponding device configurations of ITO/PEDOT:PSS/polymer: PC_71_BM/MoO_3_/Ag and ITO/ZnO/polymer:PC_71_BM/PDINO/Al in order to answer why introducing fluorine into the electron-withdrawing BT in PBDT-TVT-FBT improved device performance. These hole mobility (*μ*_h_) and electron mobility (*μ*_e_) values were calculated according to the equation $J=\;\frac{9}{8}\varepsilon_{0}\varepsilon_{r}\mu \frac{V^{2}}{L^{3}}$ [86,91,92]. The thickness values of blend films for PBDT-TVT-BT and PBDT- TVT-FBT were 110 and 112 nm for hole-only devices, and 107 and 117 nm for electron-only devices (Tables S3 and S4), respectively. The *J–V* characteristics and the fitting of *J*^1/2^−*V* curves under dark are shown in Figure S7 and Figure 9, respectively. As seen from Table 4, the *μ*_h_, *μ*_e_, and *μ*_h_/*μ*_e_ ratio values were 1.90 × 10^−4^, 4.51 × 10^−5^ cm^2^ V^−1^ s^−1^, and 4.21 for PBDT-TVT-BT, 3.06 × 10^−4^, 1.21 × 10^−4^ cm^2^ V^−1^ s^−1^, and 2.53 for PBDT-TVT-FBT, respectively. Evidently, the 1.61-times increased *μ*_h_, 2.68-times enhanced *μ*_e_, and more balanced *μ*_h_/*μ*_e_ values (from 4.21 to 2.53) can partially explain the reason why the fluorinated PBDT-TVT-FBT-based device yielded the slightly raised *J*_SC_ and *FF* [55,100]. The increased hole and electron mobilities produced by fluorination can be explained by the later morphology variation of the active layer.

### 3.8. Film Morphology 

**Figure 10:**
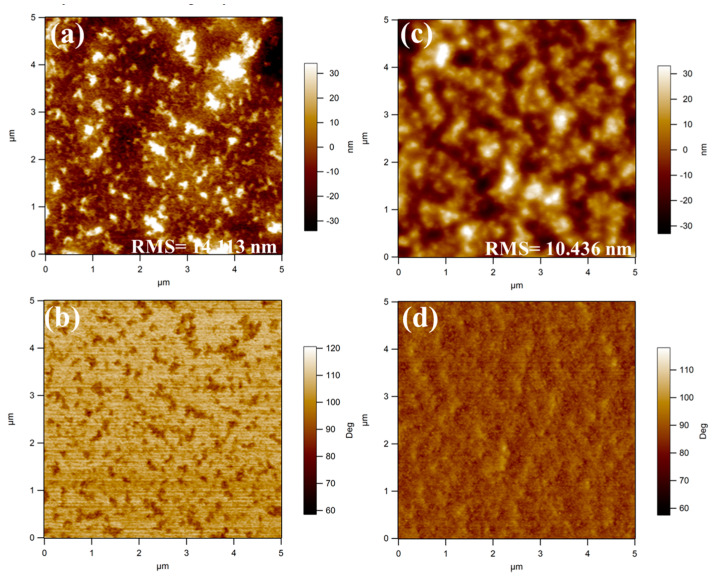
Tapping atomic force microscopy (AFM) height (top) and phase (bottom) images for photoactive layers of PBDT-TVT-BT/PC_71_BM (1:1.5, 3% DIO, **a**, **b**) and PBDT-TVT-FBT/PC_71_BM (1:1.5, 3% DIO, **c**, **d**).

**Figure 11:**
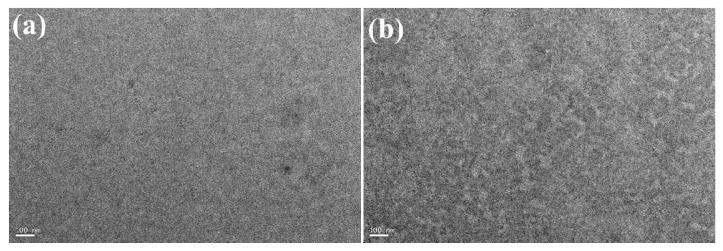
TEM bright field images of photoactive layers for PBDT-TVT-BT/PC_71_BM (1:1.5, 3% DIO, **a**) and PBDT-TVT-FBT/PC_71_BM (1:1.5, 3% DIO, **b**). The scale bar of TEM images is 100 nm.

It has been proved that the photovoltaic property was strongly dependent on the morphology of the photoactive layer in BHJ PSCs [8,21,89,100]. Thus, to deeply make a thorough inquiry into why fluorination in the PBDT-TVT-FBT-based device enhanced the PCE, the morphology of the blend films that were prepared under an exactly similar condition with as the best device was examined by AFM with a surface area of 5 μm × 5 μm. As can be seen from Figure 10, the surface morphology for PBDT-TVT-BT:PC_71_BM-based blend films showed strong aggregation with the nearest root-mean-square (RMS) roughness up to 14.113, whereas after introducing fluorine into the electron-withdrawing BT moiety in PBDT-TVT-FBT:PC_71_BM-based film, the surface became smoother and the RMS value decreased to 10.436 nm. Such variation of surface was identical with the former the result obtained from the former solid aggregation trend in XRD analysis. These relatively higher RMS values may be part of the reason for relatively lower *J*_SC_ in the *J*–*V* measurements. Additionally, TEM was selected to further investigate the in-depth morphology information of the photoactive layers, as depicted in Figure 11. It was found that the PBDT-TVT-BT:PC_71_BM-based device displayed the pinhole-like aggregation to some degree, while the fiber-like microstructure was observed after fluorination, which could be helpful to facilitate the exciton diffusion and exciton dissociation into the free charges as well as reduce the charge recombination, which could be part of the reason for the improved mobility and thus slightly elevated *FF* and *J*_SC_ after fluorination [8]. 

## 4. Conclusions

In summary, two highly conjugated 2D BDT-based alternating copolymers, namely PBDT-TVT-BT and PBDT-TVT-FBT, containing two ((*E*)-(4,5-didecylthien-2-yl)vinyl)-5-thien-2-yl conjugated side chains were designed to investigate the impact of fluorination on thermal stability, optoelectronic, and photovoltaic properties. Owing to selecting the highly conjugated electron-rich BDT-TVT building block and electron-deficient BT unit to build the polymer backbone, the lower bandgap of 1.60~1.69 eV and deeper *E*_HOMO_ of –5.17 ~ –5.37 eV were obtained. The narrowed absorption, deepened *E*_HOMO_, and weakened aggregation in solid film, but insignificant influence on thermal stability were found after incorporating the fluorine into BT in PBDT-TVT-FBT. Moreover, the optimized photovoltaic measurement illustrated that the 16% increased PCE from 4.50% to 5.22% was yielded after fluorination in PBDT-TVT-FBT, profiting by a synergistic 5.41% increased *V*_OC_, 5.08% enhanced *J*_SC_, and 4.74% elevated *FF*. The observed enhancement was mainly due to down-shifted *E*_HOMO_, increased hole and electron mobility as well as more balanced *μ*_h_/*μ*_e_, higher exciton dissociation rate, and improved microstructural morphology of the photoactive layer as a result of incorporating fluorine into the polymer backbone. Our work suggested that fluorination was an easy-to-implement and effectual tactic aimed at maximizing the photovoltaic performance. 

## Figures and Tables

**Table 1:** Optical and electrochemical properties of the studied copolymers PBDT-TVT-BT and PBDT-TVT-FBT.

Polymer	Solution	Film		$E_{g}^{\mathrm{opt}}$^1^ (eV)	*ϕ*_ox_ (V)	*ϕ*_red_ (V)	*E*_HOMO_^2^ (eV)	*E*_LUMO_^3^ (eV)	$E_{g}^{\mathrm{ec}}$^4^ (eV)
	λ_max_ (nm)	λ_max_ (nm)	λ_onset_ (nm)						
**PBDT-TVT-BT**	392, 648, 712	383, 663, 717	775	1.60	0.47	−1.11	−5.17	−3.59	1.58
**PBDT-TVT-FBT**	341, 373, 600, 648	333, 373, 508, 615, 663	735	1.69	0.67	−1.13	−5.37	−3.57	1.80

^1^ Optical bandgap was calculated from the onset of the film absorption ($E_{g}^{\mathrm{opt}}$ = 1240/$\lambda_{\mathrm{onset}}^{\mathrm{film}}$); ^2^ *E*_HOMO_ was calculated from oxidation potential of the copolymers (*E*_HOMO_ = −e($\varphi_{\mathrm{ox}}^{\mathrm{onset}}$ + 4.70) (eV)); ^3^ *E*_LUMO_ was calculated from reduction potential of the copolymers (*E*_LUMO_ = −e($\varphi_{\mathrm{red}}^{\mathrm{onset}}$ + 4.70) (eV)); ^4^ $E_{g}^{\mathrm{ec}}$ = *E*_LUMO_ − *E*_HOMO_ (eV).

**Table 2:** Dihedral angles for the trimer models of PBDT-TVT-BT and PBDT-TVT-FBT obtained from density functional theory (DFT) at the B3LYP/6-31G* level.

Polymer	Model Compound	Molecular Structure	Dihedral Angle (deg)
**PBDT-TVT-BT**	(BDT-TVT-BT)_3_	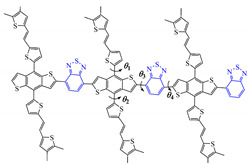	*θ*_1_ = –133.81, *θ*_2_ = –130.78, *θ*_3_ = –175.12, *θ*_4_ = –174.13
**PBDT-TVT-FBT**	(BDT-TVT-FBT)_3_	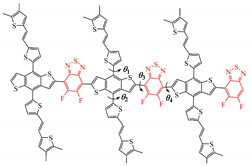	*θ*_1_ = –133.45, *θ*_2_ = –132.60, *θ*_3_ = –175.75, *θ*_4_ = –175.67

**Table 3:** The optimized photovoltaic parameters of PSCs based on PBDT-TVT-BT and PBDT-TVT-FBT.

Active Layer	Ratios/DIO	*V*_OC_ (V) ^a^	*J*_SC_ (mA cm^−2^) ^a,b^	*FF* (%) ^a^	PCE (%) ^a^	*R*_SH_ (Ω cm^2^) ^c^	*R*_S_ (Ω cm^2^) ^c^
PBDT-TVT-BT/ PC_71_BM	(1:1.5)/3%	0.74 ± 0.01	10.04 ± 0.31 (9.94)	60.57± 0.41	4.50 ± 0.29	1044.10	11.33
PBDT-TVT-FBT/ PC_71_BM	(1:1.5)/3%	0.78 ± 0.01	10.55 ± 0.45 (10.35)	63.44±0.45	5.22 ± 0.30	1560.89	9.17

^a^ The statistical results were obtained from 10 independent cells, and the ± value refers to the standard deviation; ^b^ The values in the parentheses are the integrated currents obtained from the EQE curves; ^c^ *R*_SH_ and *R*_S_ are deduced from the inverse slope at *V* = 0 and *V* = *V*_OC_ in the *J*−*V* curves under illumination.

**Table 4:** Mobilities and *μ*_h/_*μ*_e_ ratio of the studied polymers PBDT-TVT-BT and PBDT-TVT-FBT.

Active Layer	*μ*_h_^a^ (cm^2^ V^–1^ s^–1^)	*μ*_e_^b^ (cm^2^ V^–1^ s^–1^)	*μ*_h_/*μ*_e_
PBDT-TVT-BT:PC_71_BM=1:1.5, 3% DIO	1.90 × 10^−4^	4.51 × 10^−5^	4.21
PBDT-TVT-FBT:PC_71_BM=1:1.5, 3% DIO	3.06 × 10^−4^	1.21 × 10^−4^	2.53

^a^ The hole-only devices with the structure of ITO/PEDOT:PSS/polymer:PC_71_BM/MoO_3_/Ag. ^b^ The electron-only devices with the structure of ITO/ZnO/polymer:PC_71_BM/PDINO/Al.

## Supplementary Materials

The following are available online at https://www.mdpi.com/2073-4360/12/3/504/s1, Scheme S1: The synthetic route of fluorinated dibromide FBTBr_2_, Figure S1: ^1^H NMR spectrum of BTBr_2_ in CDCl_3_, Figure S2: ^13^C NMR spectrum of FBTBr_2_ in CDCl_3_, Figure S3: ^1^H NMR spectrum of BDT-TVTSn in CDCl_3_, Figure S4: ^13^C NMR spectrum of BDT-TVTSn in CDCl_3_, Table S1: Yields, GPC data and thermal properties for the studied copolymers, Table S2: The photovoltaic performance of the PSCs devices under varied fabrication processes, Table S3: Hole mobilities of the optimized devices measured by SCLC model, Table S4: Electron mobilities of the optimized device measured by SCLC model, Figure S5: UV-vis absorption spectra of copolymers PBDT-TVT-BT and PBDT-TVT-FBT dissolved in CB at various concentrations and calculation of molar absorption coefficient, Figure S6: J-V curves of PBDT-TVT-BT and PBDT-TVT-FBT with different weight ratio to PC71BM, and using 3%DIO additive and EQE spectra of corresponding PSCs, Figure S7: J-V curves of hole-only (a) and electron-only (b) devices for the PBDT-TVT-BT:PC_71_BM and PBDT-TVT-FBT:PC_71_BM.
