# Supplementary material for: Fluorination Effect for Highly Conjugated Alternating Copolymers Involving Thienylenevinylene-Thiophene-Flanked Benzodithiophene and Benzothiadiazole Subunits in Photovoltaic Application
Source: Polymers (Basel). 2020 Feb 25;12(3):504. doi: 10.3390/polym12030504 (PMC7254375; doi:10.3390/polym12030504)
Supplement: Supplementary file 1 [file polymers-12-00504-s001.pdf]

Supplementary Material

Fluorination Effect for Highly Conjugated Alternating Copolymers Involving thienylenevinylene-thiophene Flanked Benzodithiophene and Benzothiadiazole Subunits in Photovoltaic Application

Lili An[1], Yubo Huang[2], Xu Wang[2], Zezhou Liang[2,3], Jianfeng Li[2], Junfeng Tong[2]

[1] School of Chemical Engineering, Northwest Minzu University, Key Laboratory for Utility of Environment- Friendly Composite Materials and Biomass in University of Gansu Province, Lanzhou 730030, P. R. China

[2] School of Materials Science and Engineering, Lanzhou Jiaotong University, Lanzhou, 730070, P. R. China.

[3] CAS Key Laboratory of Bio-based Materials, Qingdao Institute of Bioenergy and Bioprocess Technology, Chinese Academy of Sciences, Qingdao 266101, China;

***** Correspondence: anlili2011@163.com (L. A.).

Synthesis of fluorinated $FBTBr_2$ [S1,S2]

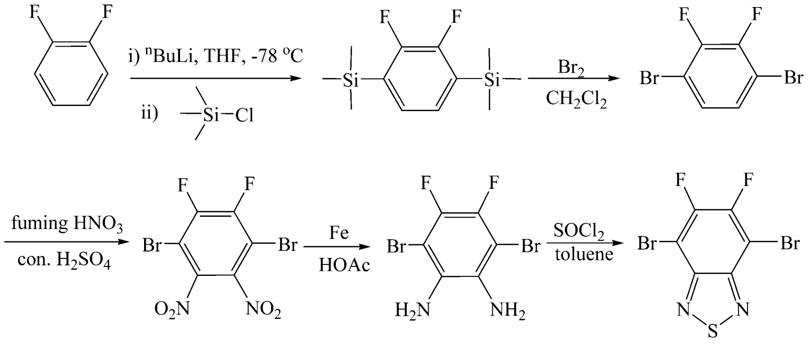

Scheme S1 The synthetic route of fluorinated dibromide $FBTBr_2$.

4,7-Dibromobenzo[*c*][1,2,5]thiadiazole ($BTBr_2$)

^{1}H NMR (400 MHz, $CDCl_3$): 7.73 (s, 2H). Alal. Calcd. for $C_6H_2Br_2N_2S$: C, 24.51, H, 0.69, N, 9.53%; Found: C, 24.40%; H, 0.58%; N, 9.61%.

4,7-Dibromo-5,6-difluorobenzo[*c*][1,2,5]thiadiazole ($FBTBr_2$)

^{13}C NMR (125 MHz, $CDCl_3$): 152.60, 150.90, 148.81, 99.32. Alal. Calcd. for $C_6Br_2F_2N_2S$: C, 21.84, N, 8.49%; Found: C, 21.70%; N, 8.59%.

2,6-Bis(trimethyltin)-4,8-bis[5-((*E*)-2-(4,5-didecylthien-2-yl)vinyl)-5-thien-2-yl]benzo[1,2-*b*:4,5-*b′*]dithiophene (BDT-TVTSn)

^{1}H NMR ($CDCl_3$, 600 MHz), δ (ppm): 7.71 (t, J = 14.4 Hz, 2H), 7.39 (d, J = 3.6 Hz, 2H), 7.13 (d, J = 3.6 Hz, 2H), 7.05 (d, J = 16.2 Hz, 2H), 6.98 (d, J = 15.6 Hz, 2H), 6.81 (s, 2H), 2.71 (t, J = 7.8 Hz, 4H) , 2.46 (t, J = 7.8 Hz, 4H), 1.65 (m, 4H), 1.54 (m, 4H), 1.39~1.28 (m, 56H), 0.89 (t, J = 7.2 Hz, 12H), 0.42 (t, J = 28.8 Hz, 18H). ^{13}C NMR ($CDCl_3$, 125 MHz), δ (ppm): 143.74, 143.42, 142.96, 139.16, 138.71, 137.94, 137.45, 131.05, 128.83, 128.67, 125.92, 122.56, 122.26, 119.84, 110.57, 32.04, 31.86, 30.82, 29.76, 29.75, 29.74, 29.70, 29.66, 29.59, 29.57, 29.47, 29.44, 28.29, 28.24, 22.82, 14.25. Anal. Calcd. for $C_{76}H_{114}S_6Sn$: C, 62.63%; H, 7.88%. Found: C, 62.44%; H, 7.71%.

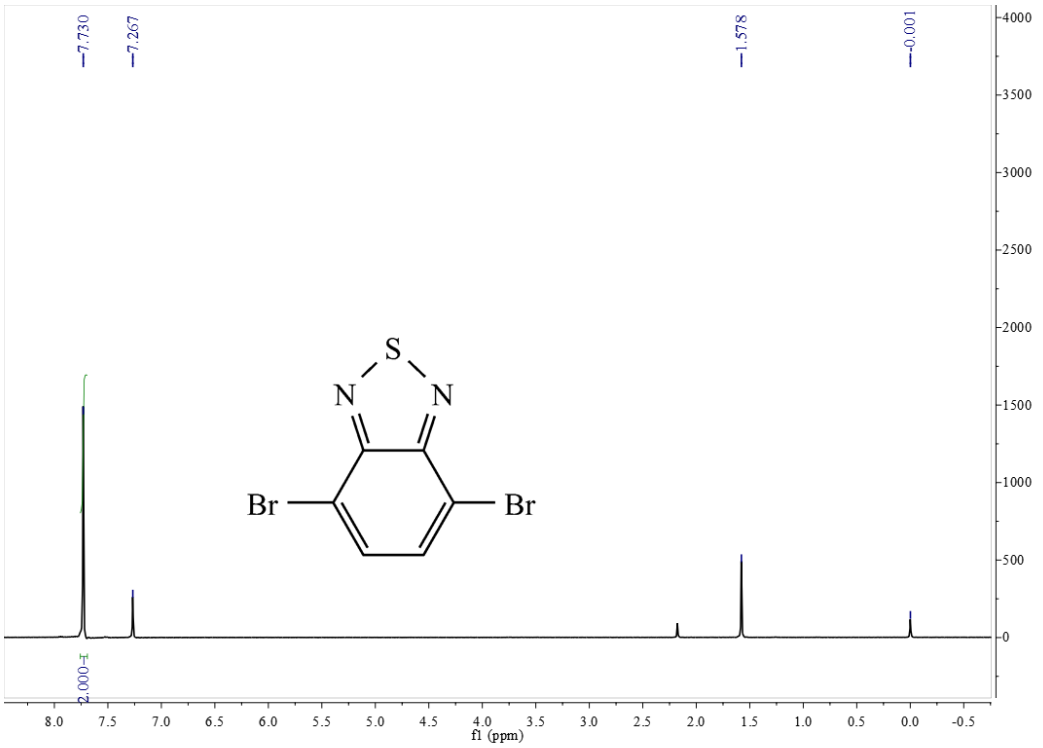

Fig. S1 ^{1}H NMR spectrum of $BTBr_2$ in $CDCl_3$.

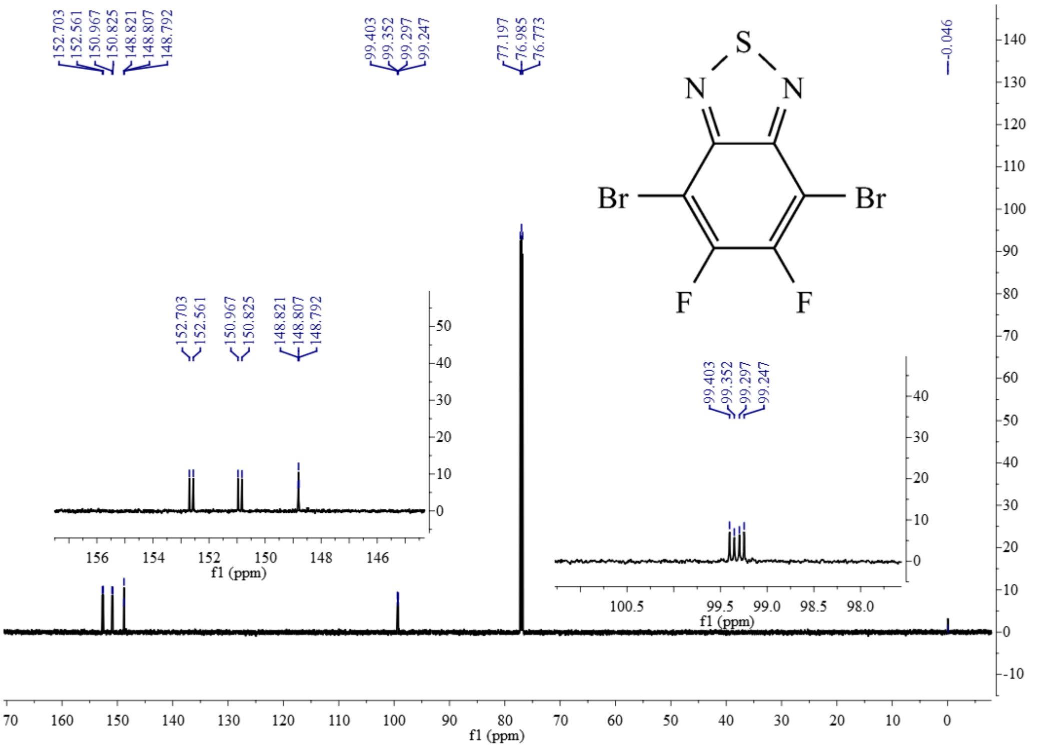

Fig. S2 ^{13}C NMR spectrum of $FBTBr_2$ in $CDCl_3$.

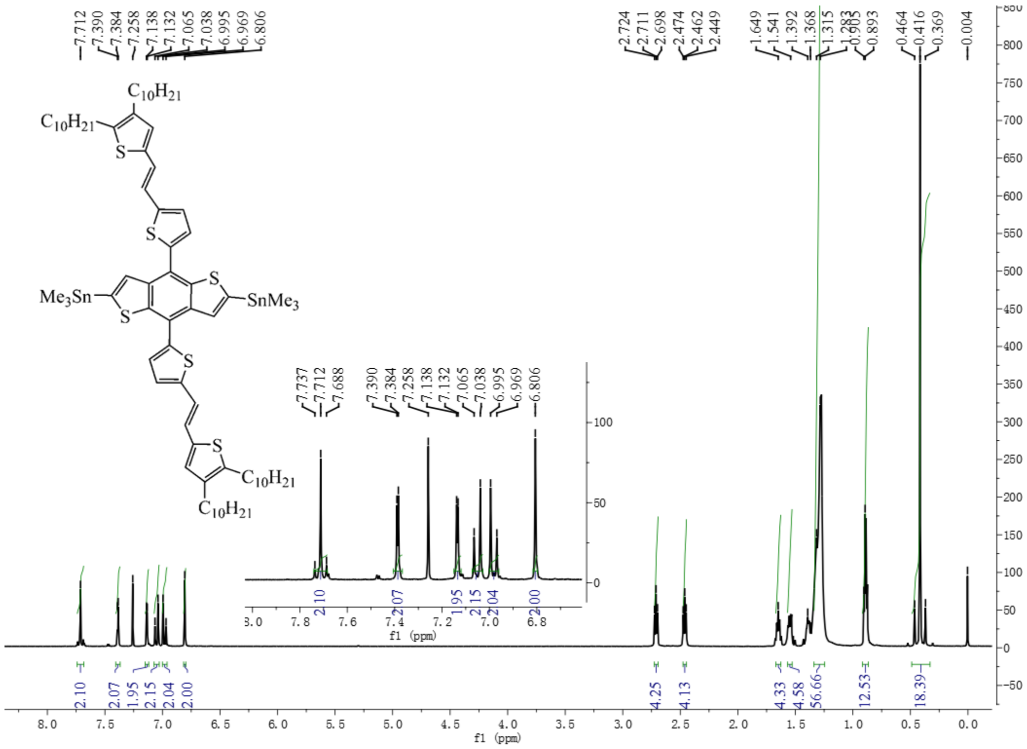

Fig. S3 ^{1}H NMR spectrum of BDT-TVTSn in $CDCl_3$.

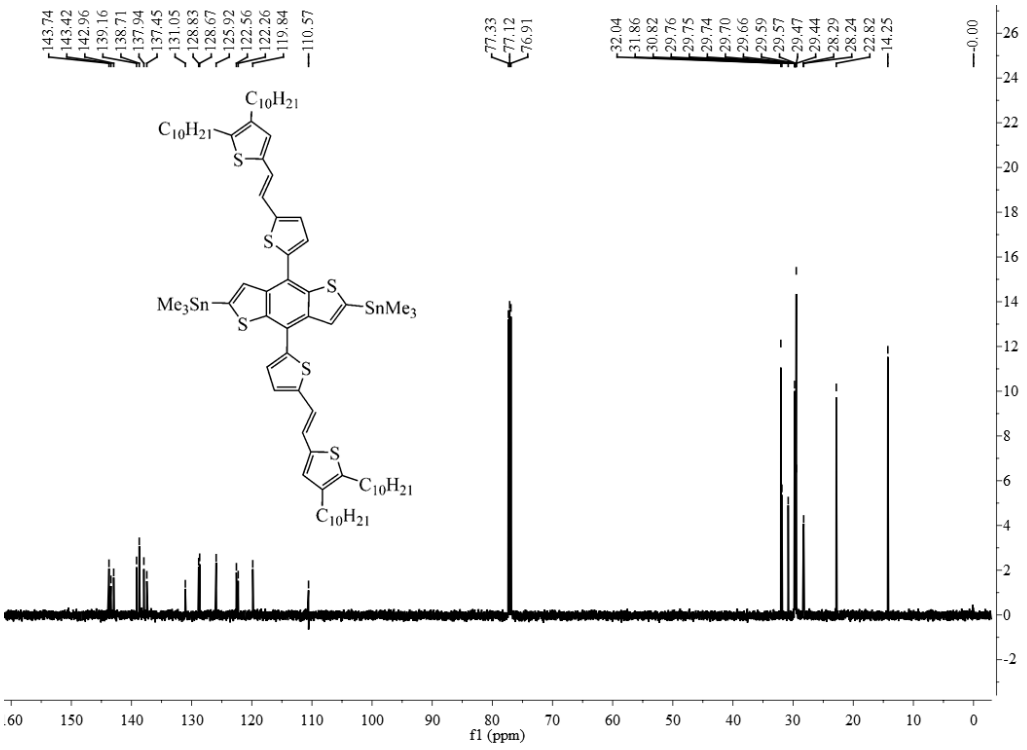

Fig. S4 ^{13}C NMR spectrum of BDT-TVTSn in $CDCl_3$.

Table S1. Yields, GPC data and thermal properties for the studied copolymers.

Polymer	Yield (%)	M_n (kDa)	M_w (kDa)	PDI	T_d (°C)
**PBDT-TVT-BT**	67.8	17.4	33.1	1.90	362
**PBDT-TVT-FBT**	69.6	18.3	38.4	2.10	362

Table S2. The photovoltaic performance of the PSCs devices under varied fabrication processes.

Active layer (w:w)	DIO	V_{OC} (V)[a]	J_{SC} (mA cm^{-2})[a,b]	*FF* (%)[a]	PCE (%)[a]	R_{SH} (Ω cm^2)[c]	R_S (Ω cm^2)[c]
**PBDT-TVT-BT**/ $PC_{71}BM$ (1:1)	0%	0.74±0.01	8.95±0.40 (8.65)	49.99±0.32	3.31±0.21	427.38	17.53
**PBDT-TVT-BT**/ $PC_{71}BM$ (1:1.5)	0%	0.74±0.01	9.29±0.45 (9.00)	56.67±0.39	3.89±0.30	819.22	13.36
**PBDT-TVT-BT**/ $PC_{71}BM$ (1:2)	0%	0.74±0.01	9.09±0.38 (8.83)	53.71±0.34	3.61±0.20	790.33	16.37
**PBDT-TVT-BT**/ $PC_{71}BM$ (1:1.5)	3%	0.74±0.01	10.04±0.31 (9.94)	60.57±0.41	4.50±0.29	1044.10	11.33
**PBDT-TVT-FBT**/ $PC_{71}BM$ (1:1)	0%	0.79±0.01	8.89± 0.38 (8.69)	55.71±0.37	3.91±0.29	978.67	13.02
**PBDT-TVT-FBT**/ $PC_{71}BM$ (1:1.5)	0%	0.79±0.01	9.44±0.41 (8.23)	58.67±0.43	4.38±0.35	1096.98	11.37
**PBDT-TVT-FBT**/ $PC_{71}BM$ (1:2)	0%	0.78±0.01	9.46± 0.45 (9.31)	56.19±0.42	4.15±0.31	981.93	12.73
**PBDT-TVT-FBT**/ $PC_{71}BM$ (1:1.5)	3%	0.78±0.01	10.55±0.45 (10.35)	63.44±0.45	5.22±0.30	1560.89	9.17

[a] The statistical results were obtained from 10 independent cells, and the ± refer to the standard deviation.

[b]The values in the parentheses are the integrated currents obtained from the EQE curves.

[c]R_{SH} and R_S are deduced from the inverse slope at $V = 0$ and $V = V_{OC}$ in the J−V curves under illumination.

Table S3 Hole mobilities of the optimized devices measured by SCLC model.

Active layer	Ratios/Additive	Thickness (nm)	Slope	μ_h (cm^2 V^{-1} s^{-1})
PBDT-TVT-BT:$PC_{71}BM$	1:1.5/3%DIO	110	20.64	1.90×10^{-4}
PBDT-TVT-FBT:$PC_{71}BM$	1:1.5/3%DIO	112	25.52	3.06×10^{-4}

Table S4 Electron mobilities of the optimized device measured by SCLC model.

Active layer	Ratios/Additive	Thickness (nm)	Slope	μ_e (cm^2 V^{-1} s^{-1})
PBDT-TVT-BT:$PC_{71}BM$	1:1.5/3%DIO	107	10.49	4.51×10^{-5}
PBDT-TVT-FBT:$PC_{71}BM$	1:1.5/3%DIO	117	15.03	1.21×10^{-4}

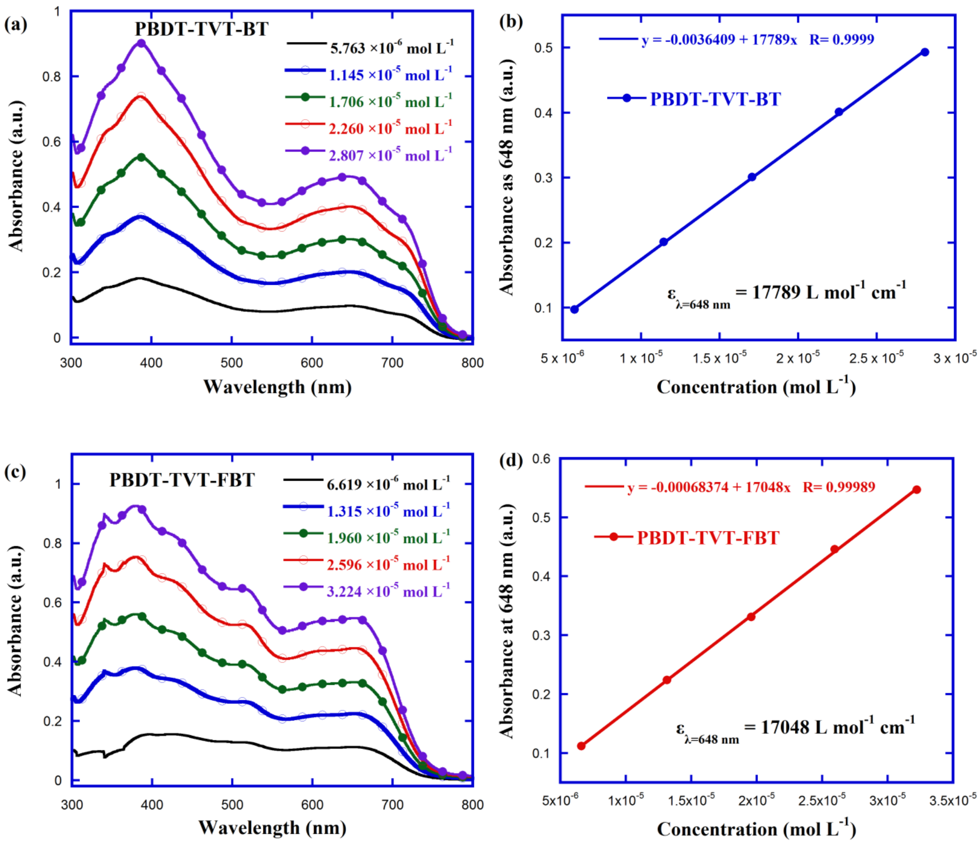

Fig. S5 UV-vis absorption spectra of copolymers PBDT-TVT-BT and PBDT-TVT-FBT dissolved in CB at various concentrations and calculation of molar absorption coefficient.

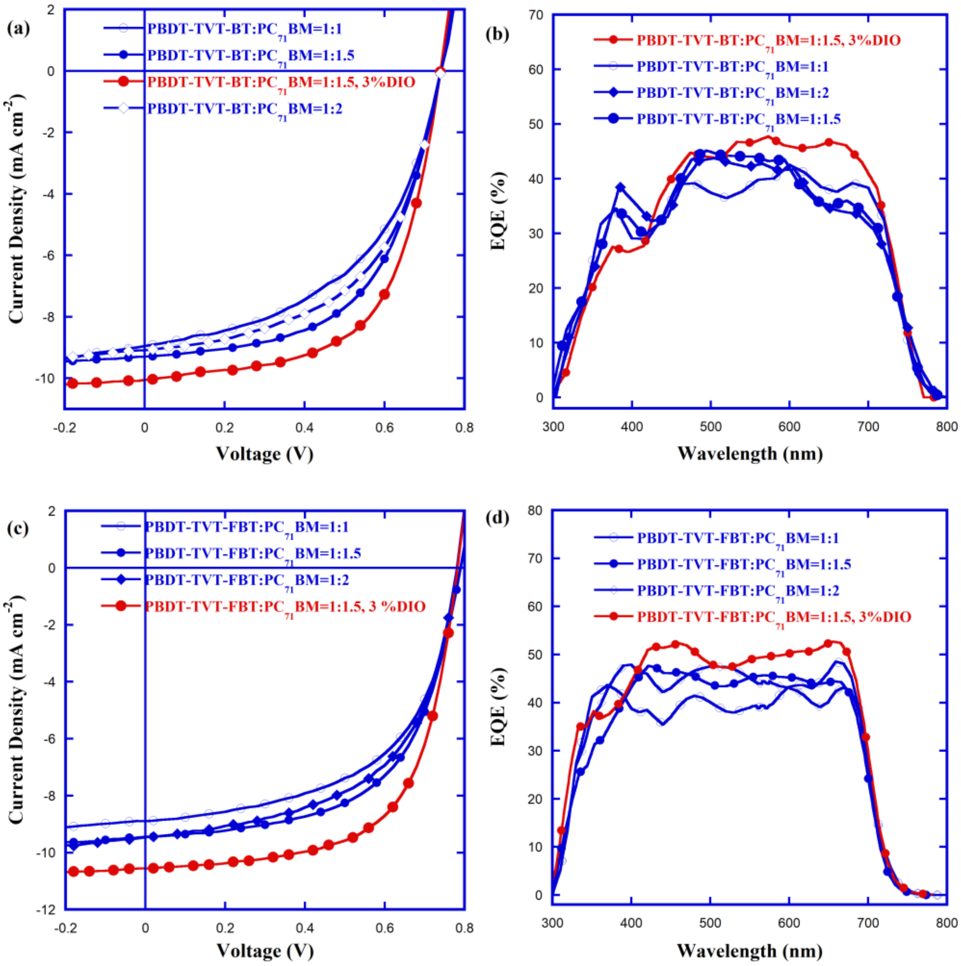

Fig. S6 *J*-*V* curves of PBDT-TVT-BT and PBDT-TVT-FBT with different weight ratio to $PC_{71}BM$, and using 3%DIO additive and EQE spectra of corresponding PSCs.

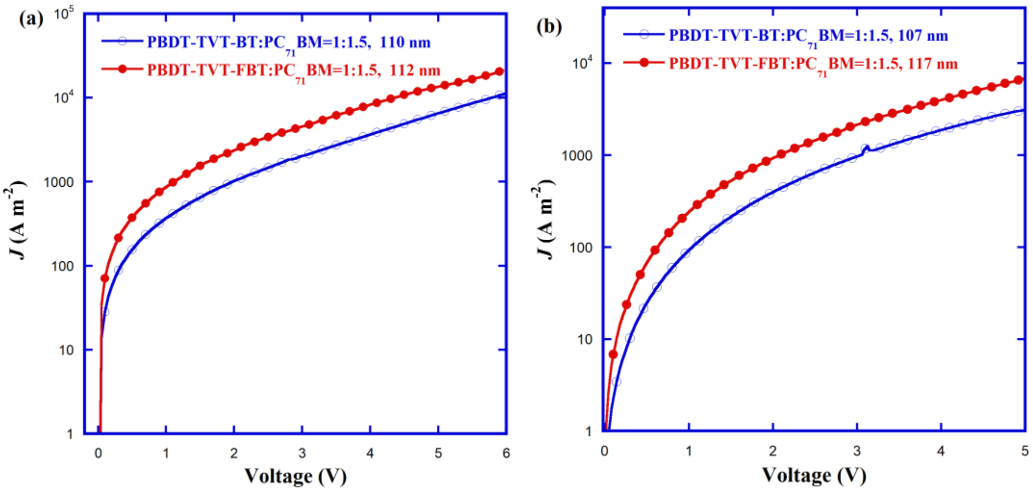

Fig. S7 *J*-*V* curves of hole-only (a) and electron-only (b) devices for the PBDT-TVT-BT:$PC_{71}BM$ and PBDT-TVT-FBT:$PC_{71}BM$.

Reference

[S1] Zhang, Y.; Chien, S.-C.; Chen, K.-S.; Yip, H.-L.; Sun, Y.; Davies, J. A.; Chen F.-C.; Jen, A.K.-Y. Increased open circuit voltage in fluorinated benzothiadiazole-based alternating conjugated polymers. *Chem. Commun.* **2011**, *47*, 11026–11028.

[S2] Guo, P.; Luo, G.; Su, Q.; Li, J.; Zhang, P.; Tong, J.; Yang, C.; Xia, Y.; Wu, H. Boosting up performance of inverted photovoltaic cells from bis(alkylthien-2-yl)dithieno[2,3-*d*:2′,3′-*d*′]benzo[1,2-*b*:4′,5′-*b*′]dithiophene-based copolymers by advantageous vertical phase separation. *ACS Appl. Mater. Interfaces* **2017**, *9*, 10937−10945.
